# Obstructed Umbilical Hernia: A Normal Presentation with Abnormal Contents

**DOI:** 10.5005/jp-journals-10018-1146

**Published:** 2016-07-09

**Authors:** Vijay P Agrawal, Nikhil S Shetty, Ashwin Narasimhaprasad

**Affiliations:** 1 Department of General Surgery, NKP Salve Institute of Medical Sciences and Lata Mangeshkar Hospital, Nagpur, Maharashtra, India; 2 Department of General Surgery, AJ Shetty Institute of Medical Sciences, Mangalore, Karnataka, India; 3 Department of Orthopedic, Ashwini Hospital, Bengaluru, Karnataka, India

**Keywords:** Cecum, Inflamed appendix, Ladd’s bands, Umbilical hernia.

## Abstract

Umbilical hernia is a common problem encountered in children. The rarity of finding cecum and appendix is probably due to the fact that the appendix is seldom found in the proximity of the umbilicus. It would, therefore, appear worthwhile to report the occurrence of cecum and an inflamed appendix with Ladd’s bands in an umbilical hernia of a child. The last case with similar presentation was presented in 1950s.

**How to cite this article:**

Agrawal VP, Shetty NS, Narasimhaprasad A. Obstructed Umbilical Hernia: A Normal Presentation with Abnormal Contents. Euroasian J Hepato-Gastroenterol 2015;5(2):110-111.

## INTRODUCTION

Umbilical hernia occurs as a result of imperfect closure or inherent weakness of the umbilical ring. It is 6 to 10 times more common in low birthweight female and black infants and 60 to 80% of premature infants present with it.^1^ Umbilical hernias are usually free from complications. We report a case of umbilical hernia with cecum and appendix with Ladd’s bands in a 5 years old child.

## CASE REPORT

**Fig. 1:**
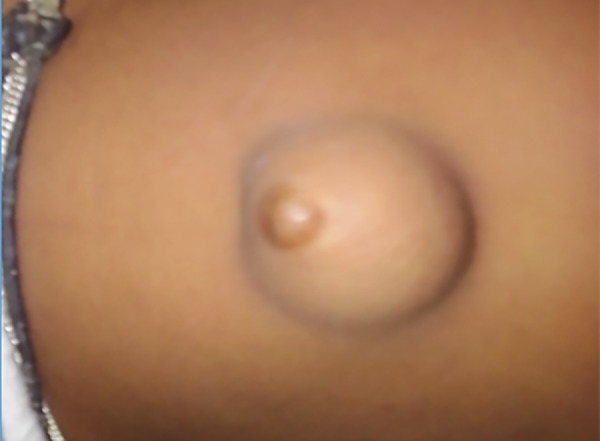
Umbilical hernia

A 5 years old female child presented in the emergency department with swelling in the umbilical region since birth, pain abdomen since 3 days associated with non bilious vomiting. On examination, the patient was calm with pulse rate of 108/minutes and respiratory rate of 22/minutes. The swelling was oval and pale pink in color at the umbilicus site. The diagnosis of obstructed umbilical hernia was made (Fig. 1).

**Fig. 2:**
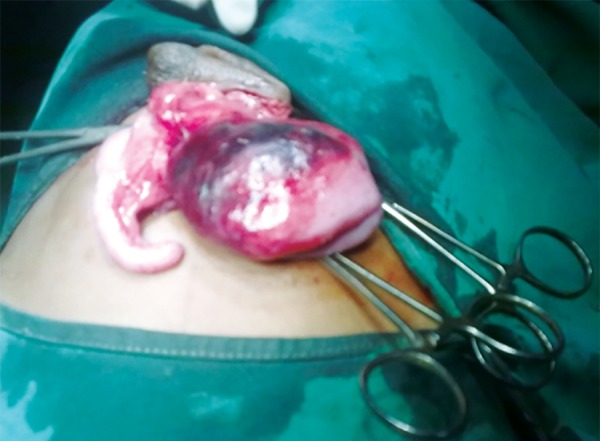
Inflamed appendix and cecum

**Fig. 3:**
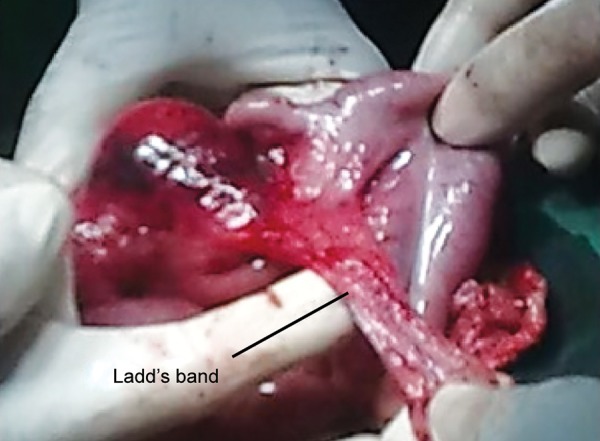
Ladd’s bands

After anesthetic clearance, the patient was operated under general anesthesia. The abdomen was opened by a horizontally placed circumferential incision. The hernia contents were cecum with inflamed appendix and presence of Ladd’s bands attached near the ileocecal junction (Figs 2 and 3).

After visceral repositioning and release of Ladd’s bands the fascial defect was repaired with a purse-string suture technique and redundant umbilical skin was excised. Postoperative period was uneventful. Urinary catheter was removed on the fourth day. Patient was allowed food orally on the third postoperative day after bowel sounds were found. Patient was discharged on the sixth postoperative day in satisfactory condition. Intravenous antibiotics were continued for 4 days followed by oral antibiotics.

## DISCUSSION

Umbilical hernia is a frequent pathology of the anterior abdominal wall in children. Race and prematurity are predisposing factors in the development of umbilical hernia. This condition is 10 times more common in African-American children than in whites, and occurs in approximately 75% of infants weighing less than 1,500 gm.^1^ The umbilical ring continues to close with time and the fascia of the defect strengthens over time leading to spontaneous closure of the defect in most children.^2^ Spontaneous closure is unlikely after the age of 3 to 5 years and in hernias with a fascial defect size larger than 1.5 cm. Therefore, it is recommended that all defects that have not spontaneously closed by age four or five should be surgically repaired.^3^ Moreover, those hernias with large fascial defects should be repaired at a much earlier age.^1^ Umbilical hernias are usually free from complications, the commonest being incarceration, followed by strangulation, only a small percentage (5%) of which become gangrenous including perforation of the content, pain and rupture following trauma.^4^ A review of literature has revealed that though there have been instances of Meckel’s diverticula or the urinary bladder being a content of umbilical hernia, there has been rarely an umbilical hernia containing cecum, appendix and associated Ladd’s bands.^5-7^ It is not clear why there was a relatively fixed cecum prolapsed through the defect in place of the mobile bowel. It is possible that the defect being more in the lower half of the umbilical ring allowed the cecum with appendix to prolapse in addition to the Ladd’s bands.

To conclude, cecum and appendix in an umbilical hernia is a very rare entity. As seen in our case, the condition is not necessarily fatal. High index of suspicion and proper evaluation are keys to success.
